# Transcatheter arterial chemoembolization plus apatinib with or without camrelizumab for unresectable hepatocellular carcinoma: a multicenter retrospective cohort study

**DOI:** 10.1007/s12072-023-10519-8

**Published:** 2023-04-03

**Authors:** Xuhua Duan, Hao Li, Donglin Kuang, Pengfei Chen, Kai Zhang, Yanliang Li, Xiang He, Cheng Xing, Haibo Wang, Yaoxian Liu, Limin Xie, Shixi Zhang, Qiang Zhang, Peixin Zhu, Honglin Dong, Jichen Xie, Hui Li, Yong Wang, Ming Shi, Guangbin Jiang, Yandong Xu, Shiqi Zhou, Chunyu Shang, Jianzhuang Ren, Xinwei Han

**Affiliations:** 1 Department of Interventional Radiology, The First Affiliated Hospital of Zhengzhou University, No. 1, East Jian She Road, Zhengzhou, Henan Province China; 2 Department of Interventional Radiology, The Third Affiliated Hospital of Zhengzhou University, Zhengzhou, Henan China; 3 Department of Interventional and Oncology, Dengzhou People’s Hospital, Nanyang, Henan China; 4 Department of Medical Imaging, Huaihe Hospital of Henan University, Kaifeng, Henan China; 5 Department of Interventional Radiology, Zhoukou Central Hospital, Zhoukou, Henan China; 6 Department of Interventional Radiology, Zhengzhou Central Hospital, Zhengzhou, Henan China; 7 Department of Interventional Radiology, Luohe Central Hospital, Luohe, Henan China; 8 Department of Interventional Radiology, Shangqiu First People’s Hospital, Shangqiu, Henan China; 9 Department of Infection, Shangqiu Municipal Hospital, Shangqiu, Henan China; 10 Department of Interventional Radiology, Anyang District Hospital, Anyang, Henan China; 11 Department of Interventional Radiology, General Hospital of Pingmei Shenma Group, Pingdingshan, Henan China; 12 Department of Interventional Radiology, The People’s Hospital of Anyang City, Anyang, Henan China; 13 Department of Interventional Radiology, The Fifth People’s Hospital of Puyang City, Puyang, Henan China; 14 Department of Interventional Radiology, The People’s Hospital of Jiaozuo City, Jiaozuo, Henan China; 15 Department of Interventional Vascular Surgery, The Second Affiliated Hospital of Hainan Medical University, Haikou, Hainan China; 16 Department of Radiology, The Second Hospital of Xingtai, Xingtai, Hebei China; 17 Department of Interventional Radiology, Suizhou Central Hospital, Suizhou, Hubei China; 18 Department of CT-MRI, Erdos Central Hospital, Erdos, Inner Mongolia China; 19 Department of Interventional Radiology, North China University of Science and Technology Affiliated Hospital, Tangshan, Hebei China; 20 Department of Interventional Radiology, Siping Central People’s Hospital, Siping, Jilin China

**Keywords:** Hepatocellular carcinoma, Transcatheter arterial chemoembolization, Apatinib, Camrelizumab

## Abstract

**Background:**

The evidence of transcatheter arterial chemoembolization (TACE) plus tyrosine kinase inhibitor and immune checkpoint inhibitor in unresectable hepatocellular carcinoma (HCC) was limited. This study aimed to evaluate the role of TACE plus apatinib (TACE + A) and TACE combined with apatinib plus camrelizumab (TACE + AC) in patients with unresectable HCC.

**Methods:**

This study retrospectively reviewed patients with unresectable HCC who received TACE + A or TACE + AC in 20 centers of China from January 1, 2019 to June 31, 2021. Propensity score matching (PSM) at 1:1 was performed to reduce bias. Treatment-related adverse events (TRAEs), overall survival (OS), progression-free survival (PFS), objective response rate (ORR) and disease control rate (DCR) were collected.

**Results:**

A total of 960 eligible patients with HCC were included in the final analysis. After PSM, there were 449 patients in each group, and the baseline characteristics were balanced between two groups. At data cutoff, the median follow-up time was 16.3 (range: 11.9–21.4) months. After PSM, the TACE + AC group showed longer median OS (24.5 vs 18.0 months, p < 0.001) and PFS (10.8 vs 7.7 months, p < 0.001) than the TACE + A group; the ORR (49.9% vs 42.5%, p = 0.002) and DCR (88.4% vs 84.0%, p = 0.003) of the TACE + AC group were also higher than those in the TACE + A group. Fever, pain, hypertension and hand-foot syndrome were the more common TRAEs in two groups.

**Conclusions:**

Both TACE plus apatinib and TACE combined with apatinib plus camrelizumab were feasible in patients with unresectable HCC, with manageable safety profiles. Moreover, TACE combined with apatinib plus camrelizumab showed additional benefit.

**Supplementary Information:**

The online version contains supplementary material available at 10.1007/s12072-023-10519-8.

## Introduction

Hepatocellular carcinoma (HCC) is one of the most common malignancies worldwide, and ranks third in terms of cancer-related death [1]. HCC is characterized by an insidious onset, a high degree of malignancy, and a high mortality rate. Most of HCC patients are in the intermediate or advanced stages when they are diagnosed, and their prognosis is poor [2]. For intermediate and advanced HCC patients, transcatheter arterial chemoembolization (TACE) is one of the most commonly used treatments [3].

TACE treatment causes necrosis in most tumor cells, leading to elevated vascular endothelial growth factor (VEGF) levels in the residual tumor [4]. Antiangiogenic agents can inhibit the proliferation and metastasis of residual tumors after TACE by blocking the hypoxia-inducible factor-1α (HIF-1α)/VEGF pathway, which may associate with an improved prognosis [4]. Apatinib is an oral tyrosine kinase inhibitor (TKI) targeting VEGFR-2, which was approved for the treatment of pretreated HCC patients [5]. A national multicenter phase 2 clinical trial in China showed that apatinib was effective and safe in the first-line treatment of advanced HCC, with a median overall survival (OS) of 9.82 months and 9.71 months in the 750 mg cohort and 850 mg cohort, respectively [6]. It is noteworthy that 27.5% of patients in the 750 mg cohort and 40% in the 850 mg cohort had previously undergone locoregional therapies [6]. Furthermore, a meta-analysis involving 14 randomized controlled trials has demonstrated that apatinib plus TACE is more effective than TACE alone for patients with unresectable HCC [7].

Programmed cell Death Protein-1 (PD-1) inhibitors restore the ability of the body’s immune system to kill tumor cells through blocking the PD-1/PD-L1 cell signaling pathway [8]. The necrotic tumor tissue after TACE will further stimulate high response to T cells, which can significantly increase the number of CD4+ and CD8+ T cells and improve the immune microenvironment [9]. Combining PD-1/PD-L1 blockades with anti-VEGF agents has revolutionized the treatment of HCC, as demonstrated first by the IMbrave150 study [10]. However, the use of atezolizumab plus bevacizumab for HCC is not covered by Chinese health insurance, putting a financial burden on patients. Besides, tremelimumab plus duruvalmab is currently unavailable in China. Thus, more treatment options are warranted. Camrelizumab, a novel immune checkpoint inhibitor (ICI), was demonstrated to be safe and effective for patients with advanced HCC in a multicenter, phase II, randomized controlled trial [11]. The RESCUE trial found that camrelizumab combined with apatinib was effective and safe in first-line and second-line treatment of advanced HCC. Among the patients included, 62.9% had previously received locoregional therapies including TACE [12]. Moreover, in a recent international phase III trial, camrelizumab plus apatinib demonstrated better survival than sorafenib for unresectable HCC in the first-line setting, with the OS of 22.1 months [13].

Therefore, patients with unresectable HCC may benefit from TACE combined with apatinib plus camrelizumab. However, the evidence of the triple combination is limited, and whether the addition of camrelizumab to TACE plus apatinib benefits the HCC patients was unknown. Thus, this retrospective study aimed to evaluate the feasibility, safety and effectiveness of the combination therapy in advanced HCC, as well as the factors associated with patients’ prognosis.

## Methods

### Study design and patients

In this retrospective study conducted at 20 centers in China, patients diagnosed with unresectable HCC who received TACE plus apatinib (TACE + A) or TACE plus apatinib and camrelizumab (TACE + AC) from January 1, 2019 to June 30, 2021 were reviewed. The study has been approved by the Ethics Committee of the First Affiliated Hospital of Zhengzhou University (Ethical approval number: SS-2020-017).

The inclusion criteria were: (1) Patients with primary non-diffuse unresectable HCC who were Barcelona Clinic Liver Cancer (BCLC) stage B/C, or those who showed recurrence following surgery; (2) Clinically or pathologically confirmed unresectable HCC with at least one measurable lesion according to the modified version of the Response Evaluation Criteria for Solid Tumors (mRECIST); (3) No systemic chemotherapy, targeted therapy other than apatinib (such as sorafenib, regorafenib), or immunotherapy other than camrelizumab (including PD-1/PD-L1/CTLA-4 inhibitors) was used; (4) aged 18–80 years old; (5) Eastern Cooperative Oncology Group Performance Status score (ECOG PS) of 0–1 within one week before treatment; (6) Less than 60% of the liver volume was occupied by tumors; (7) There are no serious comorbidities, such as hypertension, coronary heart disease, psychiatric history, or serious allergies; (8) Patients with Child–Pugh A or B (score 7), normal renal function, and normal coagulation function or be corrected after treatment; (9) Have received as least one cycle of systemic treatment (one dose of camrelizumab plus three weeks of apatinib for TACE + AC group, and three weeks of apatinib for TACE + A group). Patients received other treatment such as microwave ablation or radiofrequency ablation or changed targeted regimen before disease progression were excluded. Patients with serious comorbidities or incomplete data were also excluded.

### TACE procedure

After adequate communication with physicians, the patients were given either conventional TACE (cTACE) or drug-eluting bead TACE (DEB-TACE) as the first TACE or later TACE as per their preference. Each participated center in this study is a member of the Institute of Interventional Therapy of Zhengzhou University, and the procedures of the TACE was basically the same in each center. Generally, after local anesthesia in the right groin area, the right femoral artery was punctured using the Seldingers method, a 5-F catheter was placed, a 0.035-inch hydrophilic membrane guide wire and a 5-F RH catheter were introduced. An angiography of the trunk abdominal cavity and superior mesenteric artery was performed to determine the number, size, localization, and tumor-feeding arteries of the tumor. A microcatheter was inserted into the tumor-feeding artery, and oxaliplatin (100 mg) was slowly infused for more than 20 min. Then, 100–300 μm or 300–500 μm of drug-eluting microspheres (loaded with 40–60 mg doxorubicin or epirubicin, CalliSpheres, Hengrui Callisyn Biomedical Technology Co., Ltd., Suzhou, China) or 20–40 mg doxorubicin or epirubicin lipiodol emulsion were slowly injected until the complete disappearance of tumor staining or the development of small branches of the portal vein around the tumor. For incomplete embolization, 350–560 um polyvinyl alcohol (PVA) particles (Hangzhou Alikang Pharmaceutical Technology Co., Ltd., Zhejiang, China) or 300–500 um microspheres (Embospheres, Merit Medical, South Jordan, USA) can be added. Sheaths were pulled out and pressure bands were applied after embolization. The TACE procedure would be repeated when the tumor still had an arterial blood supply assessed by two experienced radiologists using enhanced computerized tomography (CT) or magnetic resonance imaging (MRI) and a Child–Pugh classification of A or B was confirmed.

### Systemic therapy

Treatment plans were determined by the physician’s recommendations and the patient's willingness. Hepatoprotective, analgesic, antiemetic, acid-suppressing, and other symptomatic support treatments were administered within 3–5 days after TACE. Once the condition was stable, apatinib (Hengrui Pharmaceutical Co., Ltd., Shanghai, China) was given orally at 250 mg/day. The dose of the drug should be adjusted or suspended if adverse events (AEs) of grade 3 or higher occurred during the treatment. The suspension period should not exceed 2 weeks, and the suspensions should not occur more than twice.

For immunotherapy, 200 mg camrelizumab intravenously every 3 weeks were given. Treatment-related AEs (TRAEs) such as macular papule rash, pruritus, gastrointestinal reactions, liver dysfunction, abnormal thyroid function, skin rash, immune pneumonia, reactive cutaneous capillary endothelial proliferation (RCCEP) were managed by symptomatic treatment. For serious AE, camrelizumab should be suspended or discontinued.

### Follow-up and outcomes

Following the first TACE, blood routine, liver and kidney function, coagulation function, and tumor markers were reviewed every 4–6 weeks. Imaging examinations (enhanced CT or MRI) were used to evaluate the tumor response every 6 weeks. Based on the imaging findings, two experienced radiologists assessed tumor response using mRECIST. The effectiveness outcomes included objective response rate (ORR, complete response [CR] + partial response [PR]) and disease control rate (DCR, CR + PR + stable disease [SD]), progression-free survival (PFS) and OS. PFS was defined as the time from treatment initiation to disease progression or death, whichever came first. OS was calculated as the time form treatment initiation to death of any cause.

Patients or their family members were asked about AEs, survival status, and causes of death (if applicable) after treatment by outpatient visit, WeChat, and/or telephone. The severity of AEs was recorded according to the National Cancer Institute Common Adverse Events Evaluation Criteria Version 4.03 (NCI-CTCAE 4.03).

### Statistical analysis

All data were analyzed using SPSS 26.0 software (IBM Corporation, Armonk, NY, USA). Continuous variables were reported as mean ± standard deviation (SD) or median and interquartile range (IQR), and were compared using t test or Mann–Whitney U test. Categorical variables were presented as numbers (percentages), and were compared using Chi-square or Fisher’s exact test. Time-to-event data were estimated using the Kaplan–Meier method, and compared using the log-rank test. Propensity score matching (PSM) analysis at a 1:1 ratio was performed to reduce potential bias. Age, sex, ECOG PS score, number of lesions, tumor distribution, maximum tumor diameter, hepatic vein invasion, extrahepatic metastasis, etiology, Child–Pugh stage, alpha- fetoprotein (AFP), BCLC stage were included in the PSM model. We then formed matched pairs using nearest-neighbor methods, with a caliper width of 0.05. Univariate and multivariate Cox proportional hazards regression model were conducted to determine the associated factors of OS and PFS. All baseline variables were included in the univariate analysis, and the multivariate analysis included variables with a p < 0.1 in the univariate analysis. Hazard ratios (HRs) and 95% confidence interval (CI) were determined. All statistics were two-sided, and p < 0.05 was considered statistically significant.

## Results

### Baseline characteristics of patients

**Fig. 1:**
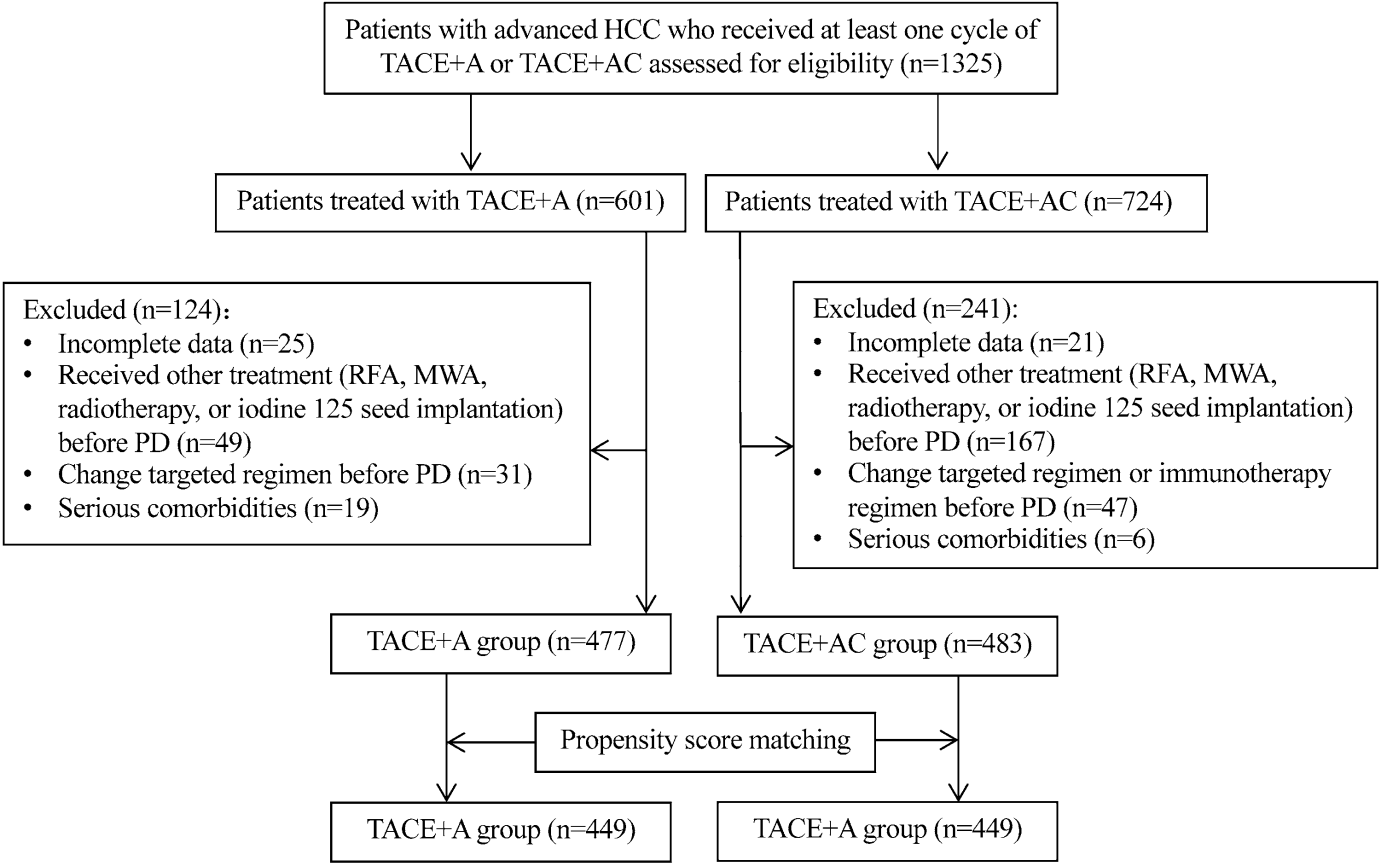
Flow diagram of patients. TACE transcatheter arterial chemoembolization, A apatinib, C camrelizumab, PD progression disease, MWA microwave ablation, RFA radiofrequency ablation

From January 1, 2019 to June 30, 2021, a total of 1325 unresectable patients from 20 centers in China were screened for eligibility. Among them, 365 patients were excluded, and finally 960 patients were included in the analysis, with 477 patients in the TACE + A group and 483 patients in the TACE + AC group (Fig. 1). Before PSM, there were no statistically significant differences between the two groups regarding sex, age, ECOG PS, number, size and distribution of tumors, etiology, Child–Pugh stage, BCLC stage (All p > 0.05). There were more patients with AFP > 400 ng/mL in the TACE + A group (p = 0.029). In the TACE + A group, 178 patients used DEB-TACE and 299 used cTACE in the first TACE, while in the TACE + AC group, 196 used DEB-TACE and 287 used cTACE in the first TACE (p = 0.142). There was no difference in the number of TACE or the duration of apatinib patients received between two groups (All p > 0.05). After PSM, there were 449 patients in each group, and the baseline characteristics were balanced between two groups (All p > 0.05, Table 1).

**Table 1:** Baseline characteristics of patients

Characteristics	Before PSM			After PSM		
	TACE + A group (n = 477)	TACE + AC group (n = 483)	p value	TACE + A group (n = 449)	TACE + AC group (n = 449)	p value
Sex, male, n (%)	382 (80.1)	399 (82.6)	0.357	367 (81.7)	372 (82.9)	0.727
Age, (years), mean ± SD	52.9 ± 9.6	52.6 ± 9.2	0.621	52.7 ± 9.1	52.7 ± 8.9	0.717
≤ 53, n (%)	238 (49.9)	247 (51.1)	0.748	227 (50.6)	227 (50.6)	> 0.999
> 53, n (%)	239 (50.1)	236 (48.9)		222 (49.4)	222 (49.4)	
ECOG PS, n (%)			0.376			0.736
0	265 (55.6)	283 (58.6)		256 (57.0)	262 (58.4)	
1	212 (44.4)	200 (41.4)		193 (43.0)	187 (41.6)	
Number of lesions, n (%)			0.305			0.541
≤ 3	184 (38.6)	203 (42.0)		178 (39.6)	188 (41.9)	
> 3	293 (61.4)	280 (58.0)		271 (60.4)	261 (58.1)	
Tumor distribution, n (%)			0.584			0.562
Bi-lobar	157 (32.9)	150 (31.1)		142 (31.6)	133 (29.6)	
Uni-lobar	320 (67.1)	333 (68.9)		307 (68.4)	316 (70.4)	
Maximum tumor diameter, cm						
Mean ± SD	12.39 ± 4.68	11.89 ± 5.06	0.112	12.27 ± 4.57	11.91 ± 4.95	0.247
≤ 10, n (%)	182 (38.2)	175 (36.2)	0.583	171 (38.1)	164 (36.5)	0.679
> 10, n (%)	295 (61.8)	308 (63.8)		278 (61.9)	285 (63.5)	
Portal vein invasion, n (%)			0.294			0.517
None	73 (15.3)	69 (14.3)		71 (15.8)	64 (14.3)	
Vp1–2	246 (51.6)	273 (56.5)		234 (52.1)	251 (55.9)	
Vp3–4	158 (33.1)	141 (29.2)		144 (32.1)	134 (29.8)	
Hepatic vein invasion, n (%)	98 (20.5)	93 (19.3)	0.675	89 (19.8)	88 (19.6)	> 0.999
Extrahepatic metastasis, n (%)	89 (18.7)	101 (20.9)	0.427	87 (19.4)	94 (20.9)	0.618
Etiology, n (%)			0.071			0.208
HBV infection	365 (76.5)	399 (82.6)		353 (78.6)	365 (81.3)	
HCV infection	45 (9.4)	27 (5.6)		44 (9.8)	27 (6.0)	
Budd-chiari syndrome	23 (4.8)	22 (4.6)		19 (4.2)	22 (4.9)	
Others	44 (9.2)	35 (7.2)		33 (7.3)	35 (7.8)	
Child–Pugh stage, n (%)			0.926			0.540
A	193 (40.5)	193 (40.0)		185 (41.2)	175 (39.0)	
B	284 (59.5)	290 (60.0)		264 (58.8)	274 (61.0)	
AFP (ng/mL), n (%)			0.029			0.729
≤ 400	162 (34.0)	198 (41.0)		162 (16,236.1)	168 (37.4)	
> 400	315 (66.0)	285 (59.0)		287 (63.9)	281 (62.6)	
BCLC stage, n (%)			0.488			0.859
B	75 (15.7)	85 (17.6)		75 (16.7)	78 (17.4)	
C	402 (84.3)	398 (82.4)		374 (83.3)	371 (82.6)	
Type of the first TACE			0.142			0.784
Drug-eluting bead TACE	178 (37.3%)	196 (40.6%)		172 (38.3)	176 (39.2)	
Conventional TACE	299 (62.7%)	287 (59.4%)		277 (61.7)	273 (60.8)	
Number of TACE						
Median (IQR)	3 (2,4)	3 (2,4)	0.089	3 (2,4)	3 (2,4)	0.891
≤ 2times, n (%)	181 (37.9)	177 (36.6)	0.727	172 (38.3)	169 (37.6)	0.837
> 2times, n (%)	296 (62.1)	306 (63.4)		277 (61.7)	280 (62.4)	
Treatment duration of apatinib, months						
Median (IQR)	6 (4,8)	6 (4,8)	0.318	6 (4,8)	6 (4,8)	0.819
≤ 3, n (%)	126 (26.4)	127 (26.3)	> 0.999	118 (26.3)	114 (25.4)	0.477
> 3, n (%)	351 (73.6)	356 (73.7)		331 (73.7)	335 (74.6)	
Cycles of camrelizumab, cycles			NA			NA
Median (IQR)	–	7 (5,10)		–	7 (5,10)	
≤ 8, n (%)	–	221 (45.8)		–	208 (46.3)	
> 8, n (%)	–	262 (54.2)		–	241 (53.7)	

PSM Propensity score matching, TACE transcatheter arterial chemoembolization, A apatinib, C camrelizumab, SD standard deviation, ECOG PS Eastern Cooperative Oncology Group Performance Status, Vp1 third branch portal vein invasion, Vp2 second branch portal vein invasion, Vp3 first branch portal vein invasion, Vp4 main portal vein invasion, HBV hepatitis B virus, HCV hepatitis C virus, AFP alpha- fetoprotein, BCLC Barcelona Clinic Liver Cancer, IQR interquartile range

### Effectiveness analysis

At the cut-off date (June 30, 2022), the median follow-up time of patients was 16.3 (range: 11.9–21.4) months. A total of 472 patients died during the follow-up, including 184 who died from disease progression, 265 who died from complications related to liver cirrhosis (178 from liver failure, 35 from gastrointestinal bleeding, 25 from septic shock caused by spontaneous bacterial peritonitis, 23 from hepatorenal syndrome, and 4 from hepatic encephalopathy) and 23 patients who died from other causes (6 from pulmonary infection, 4 from myocardial infarction, 4 from cerebral infarction, 3 from biliary tract infection, 2 from pulmonary embolism, 2 from cerebral hemorrhage, 1 from immune nephritis, and 1 from immune pneumonia).

**Fig. 2:**
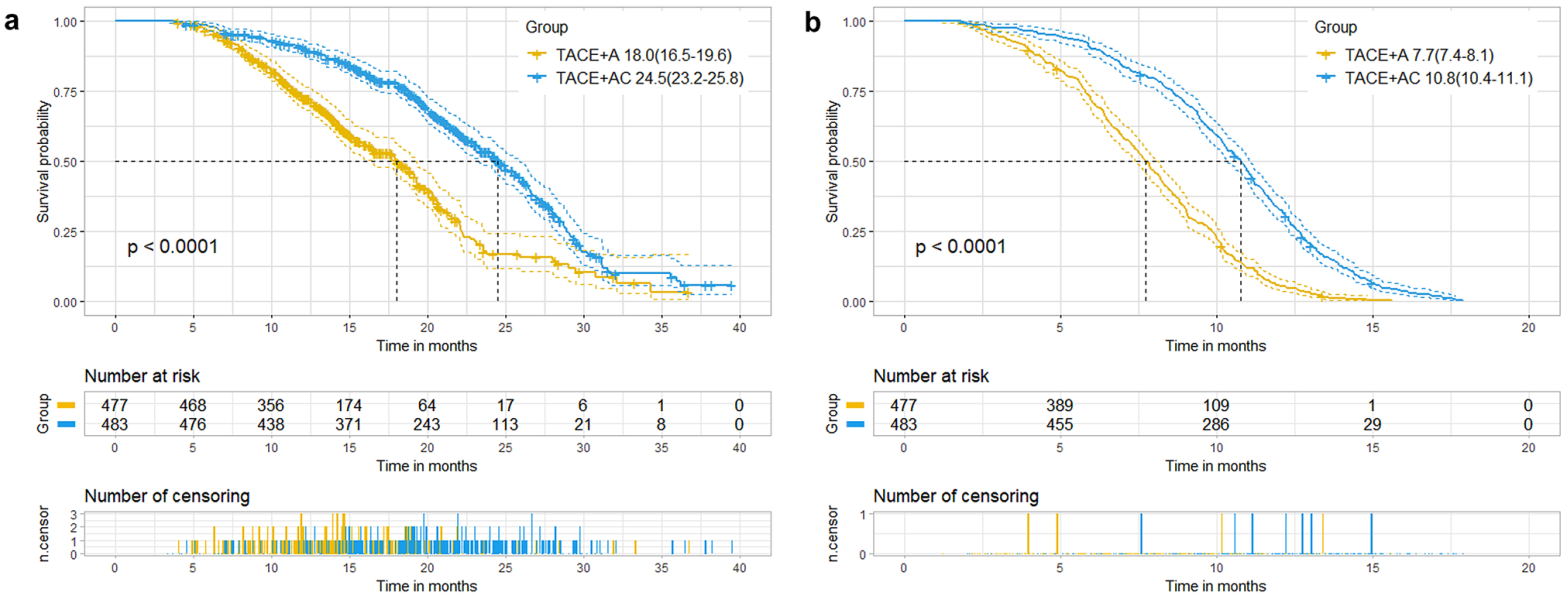
Kaplan–Meier curves of overall survival (**a**) and progression-free survival (**b**) in two groups before propensity score matching. TACE transcatheter arterial chemoembolization, A apatinib, C camrelizumab. Data were presented as median (95% confidence interval). Two groups were compared using log-rank test

**Fig. 3:**
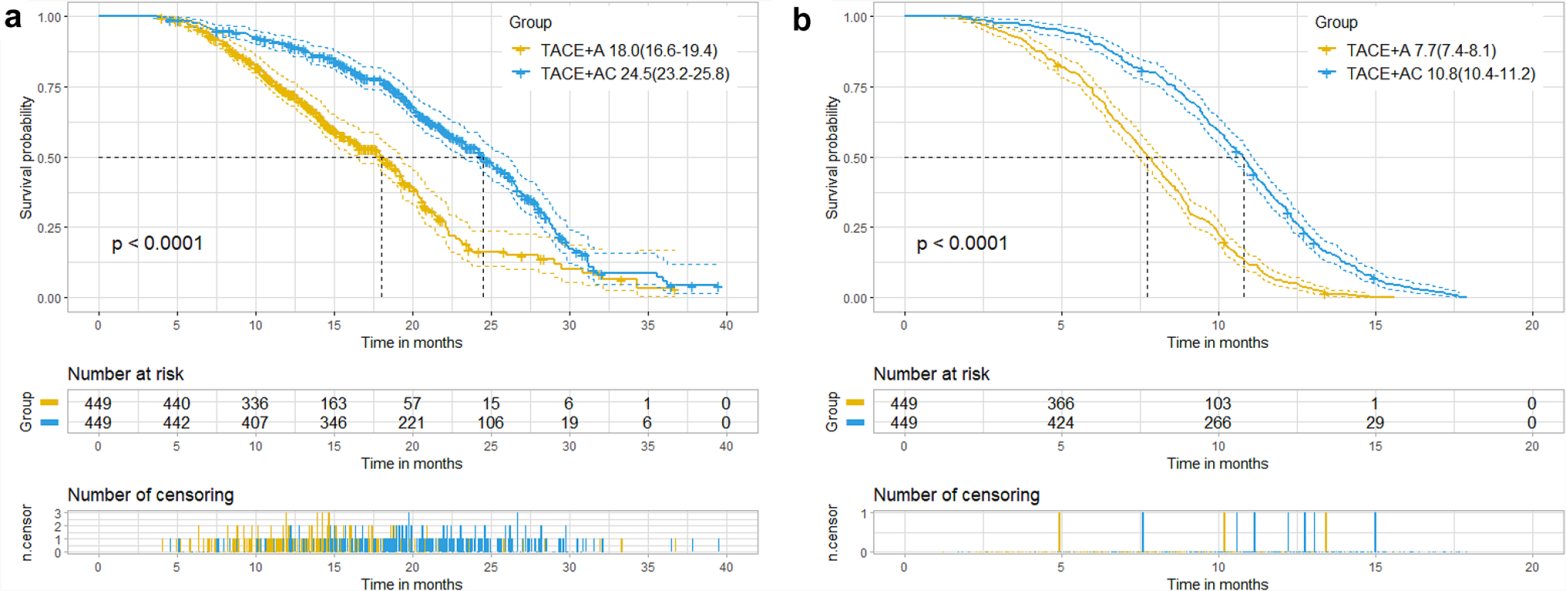
Kaplan–Meier curves of overall survival (**A**) and progression-free survival (**B**) in two groups after propensity score matching. TACE transcatheter arterial chemoembolization, A apatinib, C camrelizumab. Data were presented as median (95% confidence interval). Two groups were compared using log-rank test

Before PSM, the median OS was 18.0 months (95% CI 16.5–19.6 months) in the TACE + A group, and 24.5 months (95% CI 23.2–25.8 months) in the TACE + AC group (p < 0.001). In the TACE + A group, the median PFS was 7.7 months (95% CI 7.4–8.1 months), while in the TACE + AC group, it was 10.8 months (95% CI 10.4–11.1 months) (p < 0.001) (Fig. 2). After PSM, the TACE + AC group also showed longer median OS (24.5 vs 18.0 months, p < 0.001) and PFS (10.8 vs 7.7 months, p < 0.001) than the TACE + A group (Fig. 3).

The best of responses of patients after the first TACE in two groups were shown in Table 2. Before PSM, a higher ORR was observed in the TACE + AC group than in the TACE + A group based on mRECIST (49.5% vs. 42.8%, p = 0.003). Besides, patients in the TACE + AC group had a higher DCR than those in the TACE + A group (87.8% vs. 84.3%, p = 0.019). After PSM, the ORR (49.9% vs 42.5%, p = 0.002) and DCR (88.4% vs 84.0%, p = 0.003) of the TACE + AC group were also higher than those in the TACE + A group.

**Table 2:** Tumor response based on mRECIST between the two groups

Tumor response, n (%)	Before PSM			After PSM		
	TACE + A group (n = 477)	TACE + AC group (n = 483)	p value	TACE + A group (n = 449)	TACE + AC group (n = 449)	p value
BOR						
CR	79 (16.6)	111 (23.0)	< 0.001	75 (16.7)	104 (23.2)	< 0.001
PR	125 (26.2)	128 (26.5)		116 (25.8)	120 (26.7)	
SD	198 (41.5)	185 (38.3)		186 (41.4)	183 (38.5)	
PD	71 (14.9)	52 (10.8)		68 (15.1)	56 (10.2)	
NE	4 (0.8)	7 (1.4)		4 (0.9)	6 (1.3)	
ORR (CR + PR)	204 (42.8)	239 (49.5)	0.003	191 (42.5)	224 (49.9)	0.002
DCR (CR + PR + SD)	402 (84.3)	424 (87.8)	0.019	377 (84.0)	397 (88.4)	0.003

mRECIST modified Response Evaluation Criteria in Solid Tumors, PSM Propensity score matching, TACE transcatheter arterial chemoembolization, A apatinib, C camrelizumab, BOR best of response, CR complete response, PR partial response, SD stable disease, PD progressive disease, NE not evaluable, ORR objective response rate, DCR disease control rate

### Multivariable analysis

According to the multivariable analysis, patients in the TACE + AC group (HR = 0.44, 95% CI 0.37–0.53, p < 0.001), maximum tumor diameter greater than 10 cm (HR = 1.28, 95% CI 1.07–1.54, p = 0.008), portal vein invasion Vp3-4 (HR = 1.66, 95% CI 1.25–2.20, p < 0.001), BCLC stage C (HR = 1.28, 95% CI 1.03–1.60, p = 0.030) were independently associated with OS (Supplementary Table S1). Besides, multivariable analysis demonstrated that patients in the TACE + AC group (HR = 0.38, 95% CI 0.33–0.43, p < 0.001) and ECOG PS score of 1 (HR = 1.15, 95% CI 1.01–1.30, p = 0.039), portal vein invasion Vp3-4 (HR = 1.34, 95% CI 1.09–1.64, p = 0.005) was independently associated with PFS (Supplementary Table S2).

### Safety profiles

There was no significant difference in liver function and renal function between the two groups before and at 1 month, 6 months and 12 months after the first TACE (Supplementary Table S3). The TRAEs were shown in Table 3. For TACE related AEs, there was no significant difference in post-embolization syndrome (pain, nausea, vomiting and fever), ascites, and liver abscess. There was a higher rate of hepatic artery spasm (13.0% vs. 9.6%, p = 0.027) and thinning (32.5% vs. 21.2%, p < 0.001) during the second TACE in the TACE + AC group as compared to the TACE + A group. All the hepatic arteries spasm was significantly relieved after papaverine was administered during the second TACE. Twenty-one patients in the two groups developed liver abscesses after TACE. Among them, eleven were relieved after drainage of the abscess cavity and removal of the drainage tube; six were stabilized after anti-inflammatory treatment; four did not improve after drainage and died from liver failure.

**Table 3:** Treatment related adverse events in the two groups

Adverse events, n (%)	Any Grade				Grade 3 or 4		
	TACE + A group (n = 477)	TACE + AC group (n = 483)	p value		TACE + A group (n = 477)	TACE + AC group (n = 483)	p value
Adverse events related to TACE							
Fever	237 (49.7)	227 (47.0)	0.230		0	0	–
Pain	199 (41.7)	208 (43.1)	0.516		12 (2.5)	19 (3.9)	0.108
Gastrointestinal reaction	154 (32.3)	164 (34.0)	0.417		0	0	–
Nausea and vomiting	142 (29.8)	148 (30.6)	0.622		2 (0.4)	3 (0.6)	0.535
Ascites	51 (10.7)	62 (12.8)	0.156		5 (1.0)	6 (1.2)	0.640
Liver abscess	8 (1.7)	13 (2.7)	0.165		4 (1.0)	8 (2.1)	0.114
Hepatic artery thinning in 2nd TACE	101 (21.2)	157 (32.5)	< 0.001		0	0	–
Hepatic artery spasm in 2nd TACE	46 (9.6)	63 (13.0)	0.027		0	0	–
Adverse events related to apatinib and/or camrelizumab							
Any adverse event	386 (80.9)	388 (80.3)	0.672		127 (26.6)	131 (27.1)	0.715
Hypertension	202 (42.3)	197 (40.8)	0.458		51 (10.7)	44 (9.5)	0.365
Hand-foot syndrome	205 (43.0)	186 (38.5)	0.044		42 (8.8)	37 (7.7)	0.330
Fatigue	178 (37.3)	167 (34.6)	0.200		0	0	–
Diarrhea	95 (19.9)	111 (23.0)	0.109		5 (1.0)	3 (0.6)	0.226
RCCEP	5 (1.0)	103 (21.3)	< 0.001		0	0	–
Mouth ulcers	85 (17.8)	94 (19.5)	0.347		0	0	–
Rash	84 (17.6)	79 (16.4)	0.433		0	0	–
Proteinuria	88 (18.4)	76 (15.7)	0.101		13 (2.7)	11 (2.3)	0.480
Abdominal pain	7 (1.5)	69 (14.3)	< 0.001		2 (0.4)	3 (0.6)	0.535
Hyperammonemia	15 (3.1)	66 (13.7)	< 0.001		0	12 (2.5)	–
Decreased appetite	73 (15.3)	62 (12.8)	0.104		0	0	–
Headache	69 (14.5)	57 (11.6)	0.070		0	0	–
Hypothyroidism	4 (0.8)	49 (10.1)	< 0.001		0	0	–
Thrombocytopenia	59 (12.4)	48 (9.9)	0.074		3 (0.6)	2 (0.4)	0.438
Gastrointestinal hemorrhage	53 (11.1)	49 (10.1)	0.455		12 (2.5)	11(2.3)	0.659
Immune-related pneumonia	0	10 (2.1)	–		0	4 (0.8)	–
Immune-related myocarditis	0	6 (1.2)	–		0	2 (0.4)	–

TACE transcatheter arterial chemoembolization, A apatinib, C camrelizumab, RCCEP reactive cutaneous capillary endothelial proliferation

Regarding apatinib and/or camrelizumab related AEs, hypertension, hand-foot syndrome, fatigue, and diarrhea were the most common AEs in both groups (Table 3). RCCEP, hyperammonemia, abdominal pain and hypothyroidism were more common in TACE + AC group than in TACE + A group (All p < 0.05). In the TACE + A group, 26.6% of patients experienced grade 3 AEs, and nine patients (1.9%) discontinued apatinib due to TRAEs. The rest of them received apatinib dose reduction or interruption, and finally relieved. No grade 4 or 5 TRAE was observed. In the TACE + AC group, the incidence rate of grade 3 or 4 TRAEs was 27.1%. Sixteen patients (3.3%) discontinued AC due to TRAEs. Five patients (1.0%) were judged to have died from AC related AEs, including three cases from hepatic encephalopathy, one case from immune pneumonitis, and one case from cerebral hemorrhage due to hypertension.

## Discussion

The combination of TACE (or hepatic arterial infusion chemotherapy), TKIs and ICIs has been shown to be effective in unresectable HCC, according to a systematic review that included 15 retrospective studies [14]. However, all of these studies suffered from small sample size, with only 22–86 patients included. Besides, no results of clinical trials in the triple combination in advanced HCC is available now. To the best of our knowledge, this was the largest study aimed to evaluate the feasibility of the combination of TACE, TKIs and ICIs on unresectable HCC. Our results showed that both TACE plus apatinib and TACE combined with apatinib plus camrelizumab were feasible for patients with advanced HCC. In addition, TACE plus apatinib and camrelizumab was associated with a better prognosis.

For patients with intermediate to advanced stage HCC, it is equally important to preserve liver function as to achieve a response in order to prolong survival, and TACE is widely used as an important treatment for such patients [15]. According to the Chinese guidelines for the management of primary liver cancer [16], TACE is recommended for patients with China liver cancer (CNLC) stage IIb-IIIa and some patients with CNLC stage IIIb, which included patients with BCLC stage C and some BCLC stage B cases (unresectable HCC for many reasons, such as multiple nodules with four or more lesions, or the patient refuses surgery). Besides, the TACE procedure can also be used for patients whose main portal vein has been partially occluded, or who have developed abundant compensatory collateral branches or recanalized portal vein using portal vein stenting despite the complete obstruction [16]. However, patients with advanced HCC often have difficulty obtaining a satisfactory outcome with TACE alone. The combination of systematic treatment may further improve patients’ outcome [14].

Using anti-angiogenic targeted agents and ICIs together results in synergistic effects. Inhibition of VEGF increases cytotoxic T lymphocyte infiltration and reduces regulatory T lymphocyte infiltration, thus providing a more favorable immune microenvironment for ICI antitumor activity [17]. Following TACE, the tumor tissue necrosis released tumor antigens, and hypoxia increased tumor cell PD-L1, permitting the use of ICIs after TACE [18, 19]. A retrospective study found that compared with TACE plus sorafenib, the addition of ICIs showed increased ORR (54.6% vs. 34.5%), DCR (81.82% vs. 55.17%), OS (23.3 months vs. 13.8 months) and PFS (16.26 months vs. 7.30 months) [20]. Our study found similar tumor responses and OS in the TACE + AC group, but a significantly shorter PFS than in the TACE and sorafenib plus ICIs, which may be due to the higher tumor burden and higher proportion of patients with BCLC-stage C in our study. According to another single-center retrospective study, TACE and lenvatinib plus PD-1 inhibitors showed prolonged OS (16.9 months vs. 12.1 months), PFS (7.3 months vs. 4.0 months) and better ORR (56.1% vs. 32.5%), DCR (85.4% vs. 62.5%) than TACE plus lenvatinib in advanced HCC, which was consistent with our results [21].

In advanced HCC, TACE is often combined with antiangiogenic targeted drugs such as apatinib to improve its efficacy. A retrospective analysis showed that TACE + A significantly improved PFS (7.0 vs. 2.5 months) and OS (17.0 vs. 7.0 months) than TACE alone [22]. Although there are more BCLC-stage C patients in the TACE group in our study than that study (84.3% vs. 78.6%) [22], OS and PFS remained at 18.0 months and 7.7 months, respectively, which demonstrated that TACE + A can improve the prognosis of intermediate and advanced HCC. It was found in a single-center retrospective study that TACE + AC improved ORR from 17.3 to 42.9%, DCR from 57.7 to 85.7%, and OS from 13.1 to 24.8 months in comparison with apatinib plus camrelizumab (A + C) [23]. Our study also showed that TACE + AC showed better OS, PFS, ORR, and DCR than TACE + A, which suggested that TACE plus apatinib and camrelizumab may provide additional benefit.

Based on the results of the RESCUE study, the PFS of A + C in the first and second-lines for advanced HCC was 5.7 months and 5.5 months, and the ORR was 34.3% and 22.5%, respectively [12]. In that study, all patients were with Child–Pugh stage A; 62.9% of patients in first-line treatment, and 77.5% of patients in second-line treatment received previous interventional therapy. All patients in our study did not receive any local or other systematic treatment before TACE, and the proportion of BCLC-stage C patients and HBV patients was similar to that in the RESCUE study [12]. Therefore, TACE + AC and TACE + A may be superior to A + C in first-line and second-line treatment of HCC, which should be further studied.

Although the number of TACE sessions, the duration of apatinib treatment and the cycles of camrelizumab used were not included in the multivariate regression model due to the potential multicollinearity, a single-center study on TACE in unresectable HCC conducted by Shi et al. [24] showed that multiple cycles of TACE treatment (HR 0.65, 95% CI 0.47–0.91, p = 0.011) were an independent protective factor for PFS, while combined portal vein invasion (HR 5.74, 95% CI 3.34–9.85, p < 0.001) was an independent risk factor for OS. In Ju’s study Ju et al. [25], larger tumor size (HR = 1.01, 95% CI 1.00–1.02, p = 0.005) was an independent risk factor affecting OS, while macrovascular invasion (HR = 2.193, 95% CI 1.08–4.44, p = 0.029) was associated with PFS, respectively. These results were basically consistent with our study. This may suggest that with the introduction of immunotherapy for unresectable HCC, the evaluation of tumor load (such as tumor number, size, distribution, etc.) becomes more important, which primarily determines the timing and strategy of combination of TACE, TKIs and ICIs.

Prior to surgery and at 1, 6 and 12 months after the first TACE, there were no significant differences in liver function and renal function between the two groups. Hypertension, hand-foot syndrome, fatigue, and diarrhea were common TRAEs in both treatment groups, which were common toxicities of apatinib and camrelizumab reported previously [12, 26]. The incidence of hyperammonemia in TACE + AC group was also higher than that in TACE + A group. It is likely due to the increased liver damage caused by triple combination in the TACE + AC group, and the blood–brain barrier injury caused by the injury of brain endothelial cells by VEGFR receptor inhibitors [27]. Consequently, blood ammonia should be tested routinely and patients who have high blood ammonia should receive blood ammonia lowering therapy. During the second TACE treatment, tumor arteries were significantly sparser in both groups compared to the first TACE treatment, which may be related to normalization of tumor arteries caused by targeted antiangiogenetic agents [28]. However, the normal hepatic artery branches and hepatic arteries were also thinner and more prone to spasticity than those in the first TACE, most likely due to the same reasons. Additionally, abdominal pain is common in this study and RESCUE [12], which may be related to gastrointestinal ischemia caused by visceral artery contraction following target and immune therapy.

ICIs was able to enhance the anti-tumor activity of T cells, but may also affect the normal immune system, resulting in immune-related AEs [29]. RCCEP and immune-related diseases are unique side effects of camrelizumab [30]. While incidence of RCCEP in the TACE + AC is significantly higher than that in TACE + A group, previous studies have demonstrated that the incidence of RCCEP in combination with apatinib is significantly lower than that in camrelizumab alone [31]. Previous studies have shown that TACE increases the number and activity of CD4+ and CD8+ T cells by stimulating necrotic tumor tissue, which promotes an improvement in the immune microenvironment [9]. Furthermore, TACE has been shown to cause immunogenic cell death as part of immunomodulation [32]. In addition, TACE was linked to an increase in pro-inflammatory cytokines such as IL-6 in the first week following TACE, which was associated with the development of hepatitis, while Th-2 associated cytokines were increased 2 months after TACE, indicating an immune-suppressive environment [33]. There was a favorable effect of TACE on immune modulation, which could enhance immunotherapy. However, the effect of TACE on the immune-related AEs was unknown. In a retrospective study of TACE + AC vs. AC conducted by Ju et al. [23], there was no significant difference in the incidence of immunotherapy related AEs between two groups, except for the gastrointestinal reaction. Based on a previous retrospective study, camrelizumab was associated with an overall AE incidence of approximately 69.2–75% [34]. Besides, Guo et al. found that 75% of patients in the TACE plus camrelizumab group had at least one AE associated with camrelizumab, and no patient developed serious AE [35]. The total incidence of AE in a prospective study with TACE and camrelizumab was 90.1%, and most of them were grade 1–3 [36]. In our study, the rates of immunotherapy-related AE (such as RCCEP) were also comparable to those seen in the Ju study's AC or TACE + AC cohort. However, the role of TACE in mediating immune-related AEs needs to be explored further in prospective studies.

In the RESCUE study [12], the rate of grade 3 and above TRAEs was 77.4% (147/190); the rate of serious TRAEs was 28.9% (55/190); 12% (23/190) patients terminated the A + C treatment due to TRAE, and 2 patients (1.1%) experienced TRAE related deaths. In this study, grade 3 or higher TRAEs were 27.1% in the TACE + AC group; 16 patients (3.3%) discontinued A + C treatment due to TRAE, and 5 patients (1.0%) experienced TRAE-related death events. There was a lower incidence of TRAEs, TRAEs with grade 3 or higher, and TRAE-related deaths in the TACE + AC group in this study, which may be due to the lower camrelizumab dose than that in the RESCUE study (200 mg once every 2 week) [12].

This study suffered from some limitations. First, the treatment procedure of apatinib and camrelizumab were basically the same in different centers, while the management of AEs caused by these two agents may be different. Second, both DEB-TACE and cTACE were allowed in this study, and the proportion of DEB-TACE and cTACE selected by different centers varied. However, the influence of two TACE procedures on the results was not evaluated in this study. Third, quality of life is quite important for patients with malignancies received different treatments, while our study was unable to collect these data due to the retrospective nature. Therefore, further randomized controlled trials are warranted to confirm our results.

## Conclusion

In conclusion, both TACE plus apatinib and TACE combined with apatinib plus camrelizumab were feasible in patients with unresectable HCC, with manageable safety profiles. Moreover, TACE plus apatinib and camrelizumab showed additional benefit compared to TACE plus apatinib.

## Supplementary Information

Below is the link to the electronic supplementary material.

Supplementary file1 (DOCX 24 KB)

## Data Availability

Data are available from the corresponding authors upon reasonable request.
