# Supplementary material for: Transcatheter arterial chemoembolization plus apatinib with or without camrelizumab for unresectable hepatocellular carcinoma: a multicenter retrospective cohort study
Source: Hepatol Int. 2023 Apr 3;17(4):915–26. doi: 10.1007/s12072-023-10519-8 (PMC10386927; doi:10.1007/s12072-023-10519-8)
Supplement: Supplementary file 1 — Supplementary file1 (DOCX 24 KB) [file 12072_2023_10519_MOESM1_ESM.docx]

Supplementary Table S1. Univariate and multivariate Cox analysis of factors associated with overall survival.

| Variables | Univariate analysis | | Multivariate analysis | |
| --- | --- | --- | --- | --- |
|  | HR (95%CI) | P | HR (95%CI) | P |
| Group (TACE+AC vs. TACE+A) | 0.45 (0.38-0.54) | <0.001 | 0.44 (0.37-0.53) | <0.001 |
| Maximum tumor diameter (>10cm vs. ≤10cm) | 1.21 (1.01-1.45) | 0.042 | 1.28 (1.07-1.54) | 0.008 |
| Portal vein invasion |  |  |  |  |
| None | Reference |  | Reference |  |
| Vp1-2 | 0.93 (0.71-1.21) | 0.589 | 0.89 (0.68-1.16) | 0.375 |
| Vp3-4 | 1.59 (1.21-2.11) | 0.001 | 1.66 (1.25-2.20) | <0.001 |
| BCLC stage (C vs. B) | 1.28 (1.03-1.61) | 0.029 | 1.28 (1.03-1.60) | 0.030 |

HR: hazard ratio; CI: confidence interval; TACE, transcatheter arterial chemoembolization; A: apatinib; C: camrelizumab; BCLC, Barcelona Clinic Liver Cancer.

Supplementary Table S2. Univariate and multivariate Cox analysis of factors associated with progression-free survival.

| Variables | Univariate analysis | | Multivariate analysis | |
| --- | --- | --- | --- | --- |
|  | HR (95%CI) | P | HR (95%CI) | P |
| Group (TACE+AC vs. TACE+A) | 0.38 (0.33-0.43) | <0.001 | 0.38 (0.33-0.43) | <0.001 |
| ECOG PS score (1 vs. 0) | 1.14 (1.00-1.29) | 0.049 | 1.15 (1.01-1.30) | 0.039 |
| Portal vein invasion |  |  |  |  |
| None | Reference |  | Reference |  |
| Vp1-2 | 1.03 (0.85-1.24) | 0.748 | 1.10 (0.91-1.33) | 0.304 |
| Vp3-4 | 1.29 (1.06-1.58) | 0.013 | 1.34 (1.09-1.64) | 0.005 |

HR: hazard ratio; CI: confidence interval; TACE: transcatheter arterial chemoembolization; A: apatinib; C: camrelizumab; ECOG PS, Eastern Cooperative Oncology Group Performance Status.

Supplementary Table S3. Liver and kidney function before and after the first TACE

|  | TACE+A group  (n=477) | TACE+AC group  (n=483) | P value |
| --- | --- | --- | --- |
| ALT (U/L), mean±SD, (n) |  |  |  |
| Baseline | 36.8±11.8 (477) | 35.1±19.2 (483) | 0.099 |
| 1-month after TACE | 56.6±47.1 (477) | 53.7±42.7 (483) | 0.318 |
| 6-month after TACE | 76.7±42.3 (454) | 78.5±54.4 (470) | 0.574 |
| 12-month after TACE | 48.6±49.2 (284) | 45.7±34.4 (421) | 0.389 |
| AST (U/L), mean±SD, (n) |  |  |  |
| Baseline | 49.9±29.8 (477) | 46.2±31.1 (483) | 0.060 |
| 1-month after TACE | 89.5±65.7 (477) | 85.1±47.1 (483) | 0.234 |
| 6-month after TACE | 71.1±52.3 (454) | 69.6±45.1 (470) | 0.641 |
| 12-month after TACE | 50.7±29.5 (284) | 49.5±31.5 (421) | 0.606 |
| ALB (g/L), mean±SD, (n) |  |  |  |
| Baseline | 38.4±7.8 (477) | 37.5±7.9 (483) | 0.076 |
| 1-month after TACE | 34.5±7.2 (477) | 34.9±8.9 (483) | 0.444 |
| 6-month after TACE | 34.9±6.2 (454) | 35.7±7.2 (470) | 0.070 |
| 12-month after TACE | 34.8±4.9 (284) | 34.1±7.4 (421) | 0.131 |
| TBil (μmol/L), mean±SD, (n) |  |  |  |
| Baseline | 19.3±10.9 (477) | 18.6±10.7 (483) | 0.316 |
| 1-month after TACE | 22.3±19.7 (477) | 23.4±18.3 (483) | 0.370 |
| 6-month after TACE | 24.8±17.9 (454) | 23.9±15.7 (470) | 0.417 |
| 12-month after TACE | 25.5±19.6 (284) | 26.1±18.7 (421) | 0.685 |
| Scr (μmol/L), mean±SD, (n) |  |  |  |
| Baseline | 55.8±18.3 (477) | 54.8±16.9 (483) | 0.379 |
| 1-month after TACE | 62.2±14.3 (477) | 63.5±16.7 (483) | 0.195 |
| 6-month after TACE | 65.0±13.8 (454) | 66.8±15.9 (470) | 0.066 |
| 12-month after TACE | 64.1±17.4 (284) | 66.5±12.7 (421) | 0.057 |
| BUN (mmol/L), mean±SD, (n) |  |  |  |
| Baseline | 5.0±1.3 (477) | 4.9±1.3 (483) | 0.234 |
| 1-month after TACE | 4.9±2.1 (477) | 4.9±2.4 (483) | 1.000 |
| 6-month after TACE | 5.3±1.9 (454) | 5.1±1.8 (470) | 0.101 |
| 12-month after TACE | 5.4±1.7 (284) | 5.2±1.9 (421) | 0.145 |

TACE: transcatheter arterial chemoembolization; A: apatinib; C: camrelizumab; ALT: alanine aminotransferase; AST: aspartate aminotransferase; ALB: albumin; TBil: total bilirubin; Scr: serum creatinine; BUN: urea nitrogen.
